# Susceptibility and cytokine responses of human neuronal cells to multiple circulating EV-A71 genotypes in India

**DOI:** 10.1038/s41598-021-97166-x

**Published:** 2021-09-07

**Authors:** Madhu Chhanda Mohanty, Swapnil Yashavant Varose, Vinay Kumar Saxena

**Affiliations:** ICMR-National Institute of Virology, Mumbai Unit, Formerly Enterovirus Research Centre, Indian Council of Medical Research, Haffkine Institute Campus, Acharya Donde Marg, Parel, Mumbai, 400012 India

**Keywords:** Viral host response, Virus-host interactions, Viral infection, Viral infection

## Abstract

Enterovirus-A71 (EV-A71) associated Hand, foot and mouth disease (HFMD) is a highly contagious viral infection affecting children in Asia–Pacific region and has become a major threat to public health. Although several EV-A71 genotypes (C, D, and G) were isolated in India in recent years, no recognizable outbreak of EV-A71 caused HFMD, Acute Flaccid paralysis (AFP) or encephalitis have been reported so far. It is essential to study the pathogenicity or cell tropism of these Indian isolates in order to understand their tendency to cause disease. We investigated the susceptibility and cytokine responses of indigenous EV-A71 genotypes (D and G) isolated from cases of AFP and genotype C viruses isolated from cases of HFMD and encephalitis, in human cells in-vitro. Although all three EV-A71 genotypes could infect and replicate in human muscle and neuronal cells, the genotype D virus showed a delayed response in human neuronal cells. Quantification of cytokine secretion in response to these isolates followed by confirmation with gene expression assays in human neuronal cells revealed significantly higher secretion of pro-inflammatory cytokines TNF-α IL-8, IL-6, IP-10 (*p* < 0.001) in G genotype infected cells as compared to pathogenic C genotypes whereas the genotype D virus could not induce any of the inflammatory cytokines. These findings will help to better understand the host response to indigenous EV-A71 genotypes for management of future EV-A71 outbreaks in India, if any.

## Introduction

Human enterovirus A71 (EV-A71) epidemics have affected several countries in the past 40 years. EV-A71 commonly causes Hand, Foot and Mouth Disease (HFMD) in children, but can result in neurological complications such as AFP, aseptic meningitis, encephalitis and cardiorespiratory complications in severe cases. It is the second most important enterovirus of public health after poliovirus. Globally EV-A71 epidemics and outbreaks have been reported in Bulgaria, Taiwan, Singapore and China^1–4^. In China a total of 489,073 HFMD cases, including 126 fatal cases were reported in 2008 and 1,155,525 cases including 353 fatal cases were reported in 2009. Genotypic changes of EV-A71 have been observed in different places over time, with the emergence of novel genotypes or sub genotypes giving rise to serious outbreaks. Since the late 1990s, intra- and inter-typic recombination events in EV-A71 have been increasingly reported in the Asia–Pacific region^5^.

In India, first reported outbreak of HFMD was in Kerala in 2003^6^ followed by a report of cases of encephalitis in western Utter Pradesh, India^7^. Both of which were reported based on serological studies. Since 2004, every year HFMD has been causing progressively larger outbreaks in India and clinical and epidemiological patterns of the disease appear to be changing^8^. A large number of EV-A71 have been isolated from stool samples of AFP cases tested during 2007–2009 in Uttar Pradesh^9^.

ICMR-National Institute Virology, Mumbai Unit (formerly Enterovirus Research Centre, India) has reported EV-A71 isolates from cases of acute flaccid paralysis, HFMD and encephalitis in past several years in Mumbai, India. Phylogenetic analysis using complete VP1 sequences classified these isolates into different genotypes. EV-A71 isolate from a case of AFP was accepted as a new genotype D in 2001^10^, whereas EV-A71 isolates from cases of HFMD and encephalitis in Mumbai, India, classified as genotype C and a new genotype G^11^. EV-A71 strain D and G are indigenous to India and not isolated in any other countries, but so far there is no recognizable outbreak of either HFMD or meningo-encephalitis has been caused by these strains. Since several EV-A71 genotypes have been isolated in India, there is a need to explore the infectivity pattern, cell tropism and immune responses to these isolates to study their pathogenesis and disease severity. Although the pathogenesis caused by EV-A71 remains unclear, EV-A71 itself or its genotypes may be one of important determinants for the disease severity apart from the host factors. Studies on cytokine/chemokine responses are required to understand the interplay between host factors, immunopathology, and severity of EV-A71 (Indian isolates) infection.

In the present study we attempted to investigate the susceptibility of human neuronal cells to EV-A71 indigenous genotypes circulating in India and explored the cytokine responses of human neuronal cells to these genotypes in order to assess the pathogenesis.

## Material and methods

### Cells and viruses

SK-N-SH (human neuroblastoma) cells were obtained from National Centre for Cell Sciences (NCCS, Pune, India), RD-A (Human Rhabdomyosarcoma) cells were obtained from Centre for Disease Control and prevention (CDC), Atlanta. The cells were maintained in Eagles Minimum Essential Medium (MEM) (Sigma, USA) with 2 mM l-Glutamine and Earle’s Balanced Salt Solution adjusted to contain 1.5 g/L Na bicarbonate with 10% Fecal Calf Serum, 0.1 mM non-essential amino acids and 1.0 mM Na pyruvate were added in case of SK-N-SH cells.

Four different EV-A71 strains isolated from clinically different patients were provided by Dr. V. K Saxena (Table 1). The viruses were grown in RD-A cells to prepare stock viruses which were kept frozen for further use.

**Table 1:** Genotypes of EV-A71 isolated from clinically different patients from India.

EV-A71 Genotypes	Age/sex	Year	Clinical sample	Clinical condition	Diagnosis	Fever	Any other information
G	40 m/F	2011	Faeces	AFP	Not known	Y	Acute pain in all four limbs, sudden onset inability to walk, difficulty in swallowing
C^a^	9 m/M	2011	Throat swab	HFMD	HFMD	Y	Fever, rashes, HFMD, no oral lesion, no neurological symptom
C^b^	42 m/M	2012	Cerebrospinal fluid	Encephalitis	Encephalitis	Y	Loose motions, deep unconsciousness, hospitalization, speech incoherent
D	60 m/M	2003	Faeces	AFP	GBS	N	Ascending symmetrical quadriparesis, follow up 11 months with residual weakness

*AFP* acute flaccid paralysis.

^a^Referred as C (HFMD) in the text for comparison between two clinically different C genotype isolates.

^b^Referred as C (Enc) in the text for comparison between two clinically different C genotype isolates.

### Single step growth curve

The extent of virus replication was determined by single-step growth as follows. Confluent SK-N-SH cells were infected with 1MOI (Multiplicity of Infection) of each EV1 genotype. After absorption for 45 min, the monolayers were washed with three changes of PBS, incubated in MEM containing 2% FBS. The virus infected cells were frozen at − 20 °C at 1, 2, 4, 6, 8, 16, 24 h post infection (hpi). The cultures were freeze thawed 3 times and the culture supernatant harvested were centrifuged to remove cell debris. Supernatants were titrated (tenfold serial dilution) to determine virus concentration.

### Quantification of live and dead cells by crystal violet staining

The EV-A71 infected cells were fixed with 100% ice-cold methanol at different time points for 10 min. The fixed cells were stained with 0.5% crystal violet solution in 25% methanol. Then the cells were incubated for about 10 min and the stain was removed by washing in water until the dye comes off. The cells were then allowed to dry at room temperature. The Optical Density (OD) was read by ELISA reader at 570 nm.

### Quantitative real time RT-PCR analysis

Total RNA was isolated from the infected and mock treated cells using QIAGEN kit according to the manufacturer's instructions. RNA samples were tested by Real time PCR with enterovirus group specific primers^12^. The results were interpreted by analysing the Cycle Threshold (CT) values with a logarithmic amplification plot using ABI 7500 software (ABI 7500 Real-Time PCR System, Applied Biosystems, Foster City, California, USA).

### Immunofluorescence staining and confocal microscopy

SK-N-SH cells were seeded into tissue culture dishes with cover glasses. The cells were infected with either EV-A71 or mock at MOI of 1. After adsorption for 45 min, the monolayers were washed thrice with PBS to remove unadsorbed virus and replaced with 2 ml of fresh medium and incubated for 24 h. At each time point the cells were washed twice with PBS and fixed with PBS containing 4% formaldehyde for immunofluorescence assay. The fixed uninfected/infected cells on cover glasses were permeabilized (Perm buffer, BD Catalogue no. 554722) for 5 min at room temperature, washed twice with PBS and non-specific sites were blocked with 3% BSA in PBS. BSA was removed after 45 min and the cell layer was washed twice with PBS. Infected cells were treated with anti-EV-A71 monoclonal antibody (Chemi-Con Catalogue no MAB 979). 100 µl of the anti-EV-A71 monoclonal antibody (diluted to 1:1000 in PBS with 3% BSA) was used for each cover glass. The plate was then kept in the moisture chamber and incubated at 4 °C overnight. Next day the antibody treated cells were kept at room temperature for 30 min. Cells were then washed twice with PBS containing 1% BSA. Secondary antibody (goat anti mouse FITC, diluted to 1/1000) were added followed by further incubation for 30 min. Cover glasses were then washed three times with PBS and counterstained with DAPI (4, 6-diamidino-2-phenylindole) for 15 min. Then the cover glasses were mounted on microscope glass slides by using ProLong antifade mountant (Molecular Probes) and examined for localization of virus antigen by using Carl Zeiss LSM 510 Meta (63 ×) confocal microscope.

### Cytokine gene expression assays by real time PCR analysis

Total RNA was isolated from the infected and mock treated cells using the RNAqueous Kit according to the manufacturer's instructions (Ambion, Inc., USA). First-strand cDNA synthesis was performed using the High Capacity cDNA Reverse Transcription Kit (Applied Biosystems, Foster City, CA, USA). For quantitative real-time PCR analysis, TaqMan Universal Master Mix (Applied Biosystems) and ABI 7500 Real-Time PCR System (Applied Biosystems, Foster City, California, USA) was used. Primers and FAM-labelled probes were obtained from Applied Biosystems (TaqMan Assay on Demand). The following probes and primers were used: IL-6 (Hs00985639_m1), IL-8 (Hs00174103_m1), IFN-γ(Hs00989291_m1), TNF-α(Hs00174128_m1), IFN-α(Hs00265051_s1), IP-10(Hs00171042_m1), RANTES(Hs00982282_m1), MCP-1(Hs00234140_m1). The mRNA fold induction for all the genes was estimated relative to GAPDH (VICTM-dye labelled). Duplicate CT values were analysed using the comparative CT (ΔΔCT) method as described by the manufacturer.

### Multiplex cytokine assay

Multiplex cytokine analysis kits (Merck, Milliplex) were used for cytokine assays and run in duplicate according to the manufacturers’ protocol. Nine cytokines/chemokine’s, including pro-inflammatory, Th1 and Th2 associated cytokines (IL-6, IL-8, IFN-γ, TNF-α, IFN-α, IP-10, RANTES, MCP-1) were analysed in serum samples using the Luminex-100 system Version 1.7 (Luminex, Austin, TX). Data analysis was performed using the MasterPlex QT 1.0 system (MiraiBio, Alameda, CA). A five-parameter regression formula was used to calculate the sample concentrations from the standard curves.

### Statistical analysis

Students T test was performed using Sigma Plot (Sigma Plot for Windows version 10, Copyright 2006, Systat Software, Inc. Palo Alto, CA, USA) where ever applicable. One way ANOVA was performed using GraphPad Prism (GraphPad Prism 5, Version 5.01, GraphPad Software, San Diego, CA, USA).

### Ethics approval

The study does not involve human participants. EV-A71 strains ((stored biological material) used in the study were provided by Dr. V. K Saxena (Saxena et al, EID, 2015), appropriate permission were obtained to publish relevant information of the patients and retrospective use of the isolates.

## Results

### EV-A71 genotype D and G infect human neuronal cells

#### Cytopathic effects and the kinetics of cell viability

**Figure 1:**
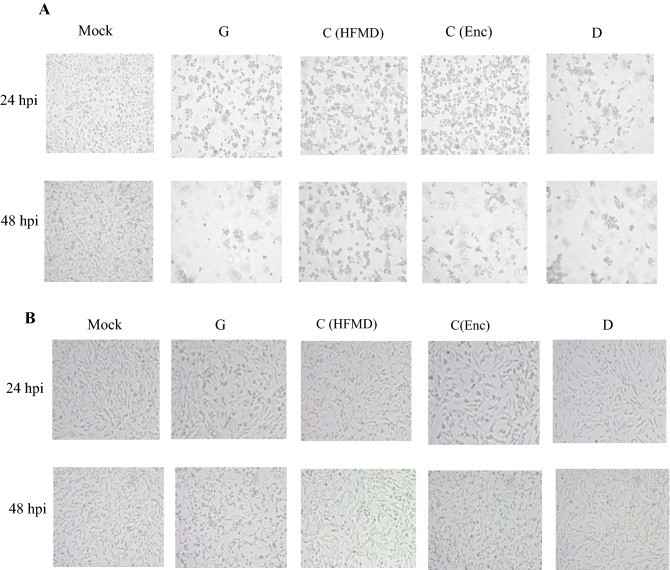
Cytopathic effect in human cells infected with the four clinically different EV-A71 isolates comprising of EV-A71 genotype G, EV-A71 genotype C(HFMD), EV-A71 genotype C (Enc) and EV-A71 genotype D (**A**) Cytopathic effect in human muscle cells (RD-A) at 24 h and 48 h post infection. (**B**) Cytopathic effect in human neuronal cells (SK-N-SH) at 24 h and 48 h post infection. Three independent experiments were performed, representative image of one experiment shown.

To understand whether the Indian genotypes infects human neuronal cells, SK-N-SH cells (human neuronal cells) and RD-A cells (human muscle cells used as positive control in the study) were infected with all the three genotypes of EV-A71 (C, D, G) with 1 MOI. The changes in both the cell lines were analysed at different hours post infection. Both the cell lines showed CPE in response to all three genotypes. RD-A cells showed observable CPE appearing at 24 hpi at MOI of 1. Genotype D and G infections in RD-A cells were more apparent at 24 hpi than C genotype. Almost 90% cells were depleted after 48 h of infection (Fig. 1A).

Compared to RD-A cells SK-N-SH cells did not show significant CPE up to 24 hpi. At 48 hpi, cells infected with G genotype started rounding up and became refractile followed by C (Enc) genotype. Cells infected with C genotype also showed initiation of CPE at 48 hpi but with very few cells as compared to D genotypes (Fig. 1B). Although at 72 h all the infected cells showed CPE, the neuronal cells infected with genotype G showed significantly higher CPE and substantial cell death G(AFP) > C(Enc) > C(HFMD) > D (AFP).

#### The kinetics of viral replication by single step growth curve

**Figure 2:**
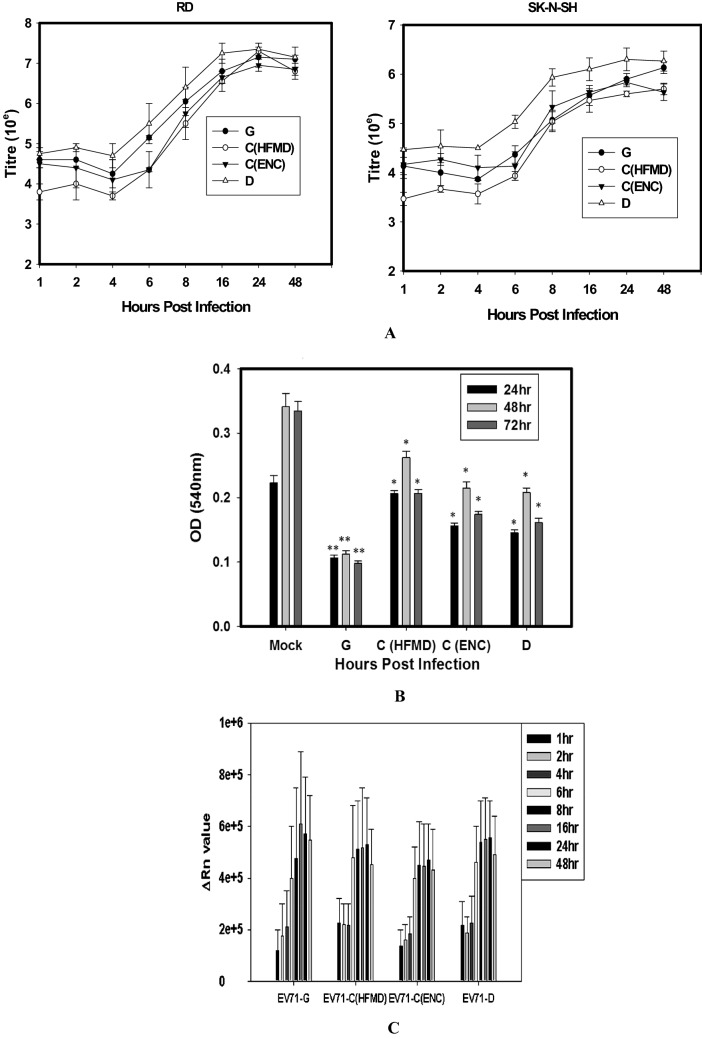
(**A**) One step growth curve of EV-A71 genotypes in RD-A and SK-N-SH cell lines. Single-step growth was measured under the infection and culture conditions described in Material & Methods. The curves were plotted based on the mean ± SE of virus titre in three independent experiments for each strain. Significant difference could be found between D versus C (HFMD) genotypes for 1 h, 4 h, 6 h, 8 h, 24 h, D versus C (Enc) genotypes for 6 h D versus G genotypes for 4 h, 6 h, 8 h, C (HFMD) versus C (Enc) for 2 h. *p* < 0.05 for all cases using student’s t test. (**B**) Cell viability of SK-N-SH cells infected with EV-A71 genotypes at different time points. SK-N-SH cells were infected with virus preparations at an MOI = 1. At 24 h, 48 h and 72 h post infection, cells were fixed and stained with crystal violet. The optical density of live cells stained in blue were measured at 540 nm in ELISA Reader. Clear wells indicated cell death due to virus infection. Three independent experiments were performed for each virus strain. The statistical analyses were performed using Sigma Plot (SigmaPlot for Windows version 10, Copyright 2006, Systat Software, Inc. Palo Alto, CA, USA) using student’s t test. *p value < 0.05; **p value < 0.01; ***p value < 0.001. All *p* values stated were referenced to the mock infected control. (**C**) Quantification of viral RNA load in SK-N-SH cells infected with different genotypes of EV71 at multiple time points post infection. Mean ± SE of QRT-PCR performed thrice. One way ANOVA test by Graph Pad prism software (GraphPad Prism 5, Version 5.01, GraphPad Software, San Diego, CA, USA) did not show any significance within the groups at a particular hour post infection.

The kinetics of replication of three genotypes in RD-A and SK-N-SH were appeared to be similar. No overall significant difference could be observed between the viral titres of three genotypes at different time points post infection (One way ANOVA). However there were significant differences observed when compared between two genotypes particularly in SK-N-SH cells (Fig. 2A). From 3 to 6 hpi the virions were slowly released into the culture media, and entered into the exponential phase from 6 to 12 hpi. At 12 hpi the rate of increase declined and the total amount of extracellular virions reached a maximum at 24 hpi. D genotype infected cells showed significantly higher viral titre at 1, 4, 6 and 24 hpi compared to the C (HFMD) genotype infected cells. Further there were significant difference in viral titre between D and G genotype infected SK-N-SH cells at 6, 8 and 24 hpi. No significant difference in viral titre could be observed in infected RD-A cell. There was one log difference in the viral titre when compared the kinetics between muscle and neuronal cell lines.

#### Live /dead cells by crystal violet staining

The cell viability of EV-A71 infected SK-N-SH cells started decreasing from 24 hpi as compared to the un-infected cell control. The OD of viable cells as assessed by crystal violet staining was comparatively lower (> 50% cell death, *p* < 0.001) in SK-N-SH cells infected with EV1-A71 G genotype corroborating the CPE data, at all the time points tested. Figure 2B confirms the infection and cell death by all three genotypes in human neuronal cells.

#### Quantification of viral RNA load by real time PCR

To confirm the above results, the level of viral RNA was measured by real time PCR at all time points of EV-A71 infection in both the cell lines. The kinetics of viral RNA in response to the three genotypes in SK-N-SH cells did not show a significant difference as tested by one way ANOVA although the increase in viral RNA in response to G genotype was higher as compared to D and C (Fig. 2C).

#### Presence of viral antigens by confocal microscopy

**Figure 3:**
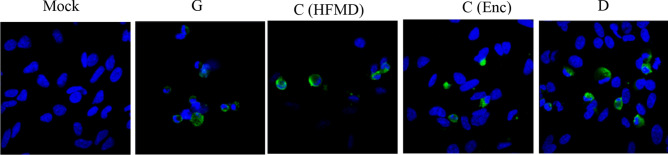
SK-N-SH cells were mock-infected or infected with EV-A71genotypes at an MOI of 5 for 24 h. Cells were probed with anti-EV71 monoclonal antibody and anti-mouse FITC, stained with DAPI and examined by confocal microscopy. EV-A71 (green), DAPI (blue) examined by confocal microscopy (Carl Zeiss LSM 510 Meta (63X) confocal microscope). Three independent experiments were performed, representative image of one experiment shown.

To confirm the presence of EV-A71 antigens in the infected neuronal cells, immunofluorescence staining of SK-N-SH cells infected with all three EV-A71 genotypes was performed. EV-A71 antigens could be observed in the cytoplasmic region of infected cells of all three types (Fig. 3).

### Cytokine/chemokine levels in EV-A71 infected human cells

**Figure 4:**
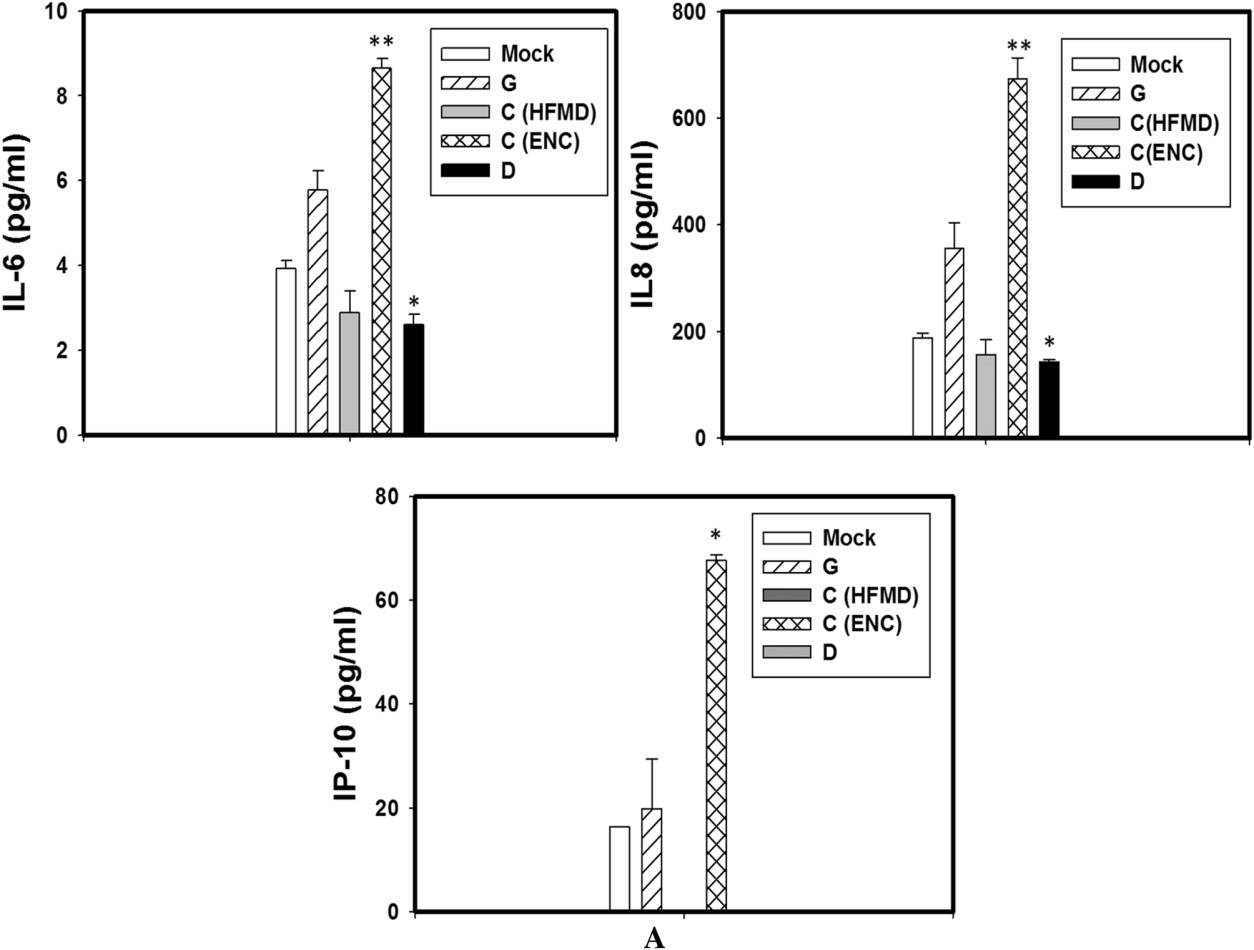

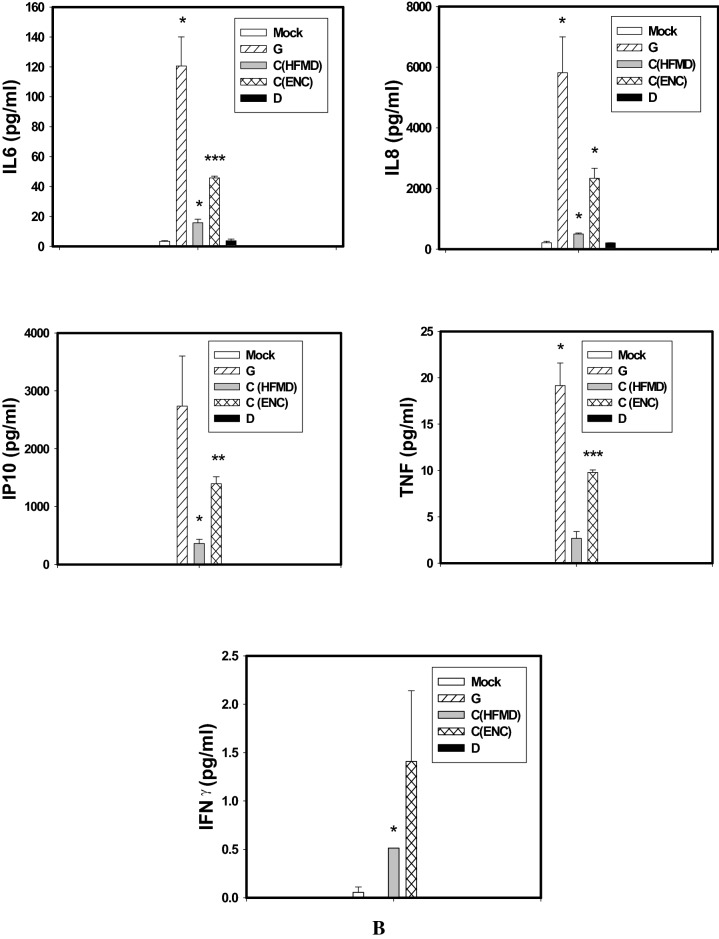

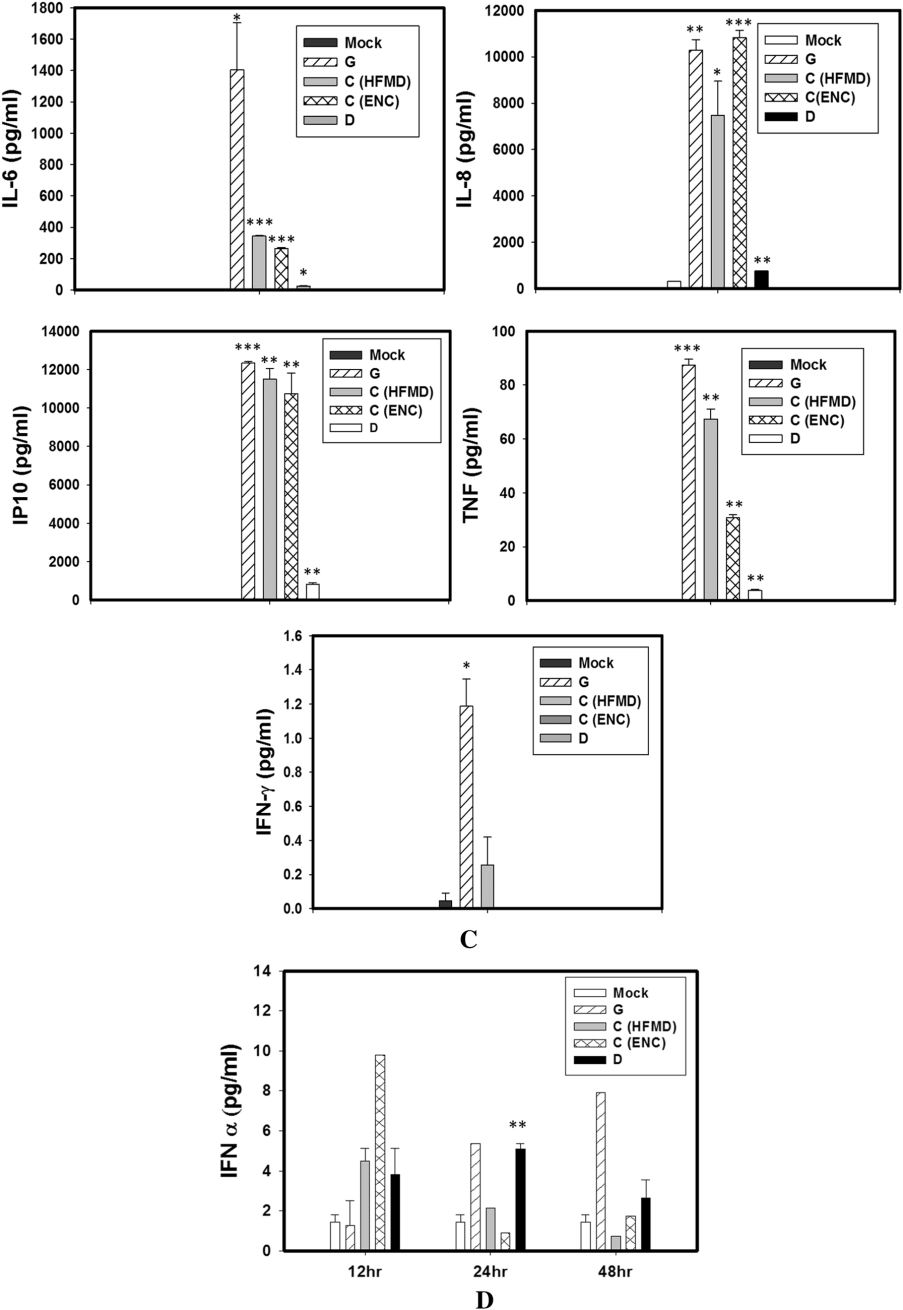
SK-N-SH cells were mock-infected or infected with EV-A71genotypes at an MOI of 1 for different time points. The cytokine and chemokine secretion profiles of human neuronal cells infected with different EV-A71 genotypes at (**A**) 12 h post infection, (**B**) 24 h post infection, and (**C**) 48 h post infection, showed significant differences between the indigenous genotypes. (**D**) RD-A cells were mock-infected or infected with EV-A71genotypes at an MOI of 1 for different time points and IFN-α level as quantified. Three independent experiments were performed for each virus strain. The statistical analyses were performed using Sigma Plot (Sigma Plot for Windows version 10, Copyright 2006, Systat Software, Inc. Palo Alto, CA, USA) using student’s t test. *p value < 0.05; **p value < 0.01; ***p value < 0.001. All p values stated were referenced to the mock infected control.

EV-A71 G and C (Enc) infected SK-N-SH cells released significantly higher amount of IL-6, IL-8 and IP-10 at all the time points tested by multiplex magnetic bead ELISA whereas no response could be seen in D (AFP) genotype infected SK-N-SH cells up to 24 hpi (Fig. 4A,B). The SK-N-SH cells infected with G genotypes elevated comparable amount of IL-6, IL-8, TNF-α and IP-10 as compared to C (Enc) genotype at 24 hpi. Genotype C (HFMD) virus could able to induce significant amount of IL-6, IL-8, IP-10 from 24 hpi onward.

At 48 hpi SK-N-SH cells infected with all four isolates [G, C (HFMD, C (Enc) and D] secreted higher amount IL-6, IL-8, IP-10 and TNF-α but the G genotypes infected cells continued to evoke higher responses than others particularly for production of TNF-α IFN-γ and IP-10 (*p* < 0.001) (Fig. 4C). Interestingly the C genotype isolate from HFMD patient induced higher amount of TNF-α than the encephalitis patient.

RD-A cells did not show any increase in inflammatory cytokines in response to any of the viruses. Rather RD-A cells showed increase in IFN-α production (Fig. 4D). At 12 hpi, elevated IFN-α levels could be seen in RD-A cells infected with all viruses except genotype G. However at 24 hpi the IFN-α levels decreased for C genotypes whereas it remained at comparatively higher level in G and D genotype infected cells.*

### Confirmation of cytokine release by EV-A71 infected cells by Real time PCR

**Figure 5:**
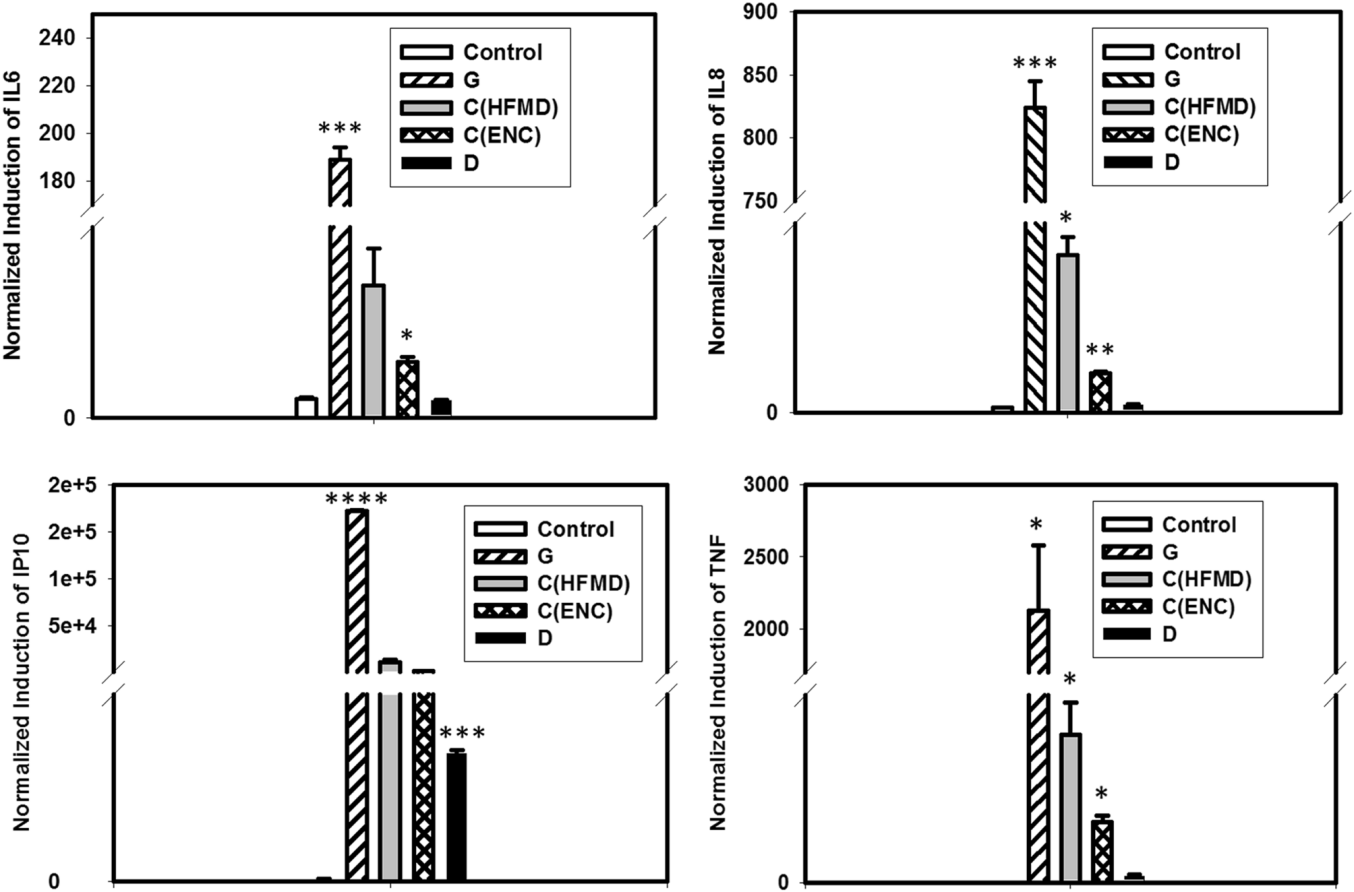
SK-N-SH cells were mock-infected or infected with EV-A71genotypes at an MOI of 1 for 24 h. The cytokine and chemokine gene expression profiles of human neuronal cells infected with different EV-A71 genotypes showed significant differences between the indigenous genotypes. Three independent experiments were performed for each virus strain. The statistical analyses were performed using Sigma Plot (SigmaPlot for Windows version 10, Copyright 2006, Systat Software, Inc. Palo Alto, CA, USA) using student’s t test. *p value < 0.05; **p value < 0.01; ***p value < 0.001. All p values stated were referenced to the mock infected control.

The RNA samples of the infected cells were extracted at 24 h post infection and the expression of related genes were measured by real time PCR. The significantly higher expression of IL-6, IL-8, IP-10 and TNF-α in SK-N-SH cells infected with EV-A71 G genotype were confirmed by gene expression (*p* < 0.001) when compared to the uninfected mock cells and also compared with other two genotypes. Further it was also confirmed that EV-A71 D genotype is unable to induce the expression of IL-6, IL-8 and TNF genes in SK-N-SH cells. Although this genotype did not produce IP-10 cytokines at 24 hpi, the expression of IP-10 gene was observed in the infected cells (Fig. 5).

From the two indigenous genotypes (D and G), infection with genotype D did not release any of the inflammatory cytokines as compared to that of the other genotypes whereas infection with genotype G showed significantly higher TNF-α IL-6, IL-8, IP-10 production in infected neuronal cells.

## Discussion

Genomic characterization of EV-A71 strains isolated globally has revealed 7 genotypes designated as EV-A71 genotypes A through G. Genotype A represents the prototype strain, genotype B consists of sub-genotypes B0–B5, genotype C consists of sub-genotypes C1–C4, genotype D is predominant type in India which has not been reported from any other country, genotypes E and F were reported from Africa, genotype G is reported in India only. Genotypes D and G may be endemic in India because they were isolated from AFP patients in wide geographic areas; however, these genotypes have not been implicated in any specific outbreaks. Moreover, the 2 genotypes appear to be indigenous to India because they have not been detected in any other country^11^. Of the total number of EV-A71 isolated at our Institute, D genotypes constitute 70–80%, C genotype 15% and G genotype 5% (Saxena et al., personal communication). Although several HFMD outbreaks have been reported in India, none have been reported to be caused by EV-A71. So far there has been no investigations conducted on whether the Indian strains are naturally attenuated or genetically resistant or have tendency to cause epidemics.

In this study we have used in-vitro cell culture methods to investigate the infectivity and immune responses to Indian strains. Four different isolates obtained from patients with different clinical symptoms were studied in two different human cell lines. Cellular tropism of the viruses were studied to ensure the extent of replication and further successful spread of the virus. Some unique characteristics were noted which may explain important aspects of the epidemiology and mechanism of pathogenesis of the indigenous strains.

Neurological virulence is one of the most severe complications responsible for death in HFMD^13^. The mechanism of the neurological complications of EV-A71 infection is poorly understood^14^. Neuronal cell tropism of all three genotypes were tested by infecting human neuronal cell lines. EV-A71 D genotype has a delayed CPE in neuronal cells, but no significant difference could be observed in cell viability of D and C genotype infected cells. The infectivity of indigenous genotypes in human neuronal cells was confirmed by confocal microscopy, qPCR, and single step growth curve. A significant difference in vial titre between D and C (HFMD) infected neuronal cells at 1 hpi was consistently observed indicating lower viral attachment or low replication rate of genotype C (HFMD) viruses to SK-N-SH cells although the viral replication was faster at later time points. In addition, D genotype infected neuronal cells showed higher viral attachment and replication at various time points as compared to other genotypes. However, it is important to note that, unlike other genotypes, no substantial pro-inflammatory cytokine expression could be detected in D genotype infected cells when tested at different time points. On the contrary the indigenous G genotype showed significantly higher CPE, cell death in human neuronal cells in comparison to the pathogenic C and indigenous D genotypes. These observations in response to genotype G indicates induction of apoptosis, autophagy and inflammatory cytokines in G infected neuronal cells as reported for EV71 infection by Lei etal, 2020; which is probably not shown by genotype D^15^.

Increasing evidence suggests that inflammatory cytokines may play a central role in HFMD derived from EV-A71 infection^16–18^. It has also been postulated that dysregulation of inflammatory responses and cellular immunity are possibly responsible for this pathogenesis^19–22^. EV-A71 infected cells produce cytokines such as TNF-α, IFN-γ, and IL-6, which play an important role in controlling viral replication and infection at the early stage. Further these cytokines activate the infected cells to release inflammatory mediators and other cytokines responsible for interfering viral replication and killing infected host cells^23^.

Interestingly, the muscle cells (RD-A) infected with all three genotypes did not show substantial inflammatory cytokine secretion indicating that these genotypes are not able to induce inflammatory cytokines in muscle cells. There are reports suggesting that EV-A71 strain ATCC VR-784 infection could trigger RD-A cells to release of TNF-α at 5 MOI, with a significant increase at 20 and 32 hpi^24^. In our study, infection of RD-A cells with the genotypes D, G and C with 1 MOI could not produce pro-inflammatory cytokines such as IFN-γ, TNF-α IL-6, IL-8, IP-10, MCP-1 as tested by Multiplex ELISA and validated by real time PCR. However, it is also reported that EV-A71 infection does not stimulate the expression of IFN-γ, ISG54, ISG56, and TNF-α in RD-A cells^25^ which supports our result. It is important to note that in our study EV71 infected RD-A cells could induce IFN-α in response to all the three genotypes at different time points including the genotype D in contrast to the report that EV71 antagonizes type I IFN signalling there by reducing IFN release^26^. IL-6 and IL-8 are considered as important markers of inflammation as they induce inflammatory responses^27^. In critically ill HFMD cases, augmentation of IL-6 and IL-8 levels in the plasma suggested high-level systemic inflammation, indicating involvement of IL-6 in EV-71 pathogenesis^28^. In cases of severe complications of the disease, anti-IL-6 antibody treatment represents a potential therapeutic approach providing protection^29^. In our study human neuronal cells infected with the pathogenic C genotype virus isolated from a case of encephalitis secreted significantly higher amount of IL-6, IL-8 and IP-10 cytokines as compared to other genotypes at 12 hpi. However, the secretion of all the cytokines (except IFN-γ) increased significantly in response to indigenous G genotype at 24 h and 48 h resulting in highly elevated cytokine secretion as compared to the pathogenic C genotypes. The cytokine secretion data has been confirmed by expression of cytokine genes which revealed significantly higher expression IL6 and IL8 genes in both C and G genotype infected human neuronal cells at later time points further confirming that the indigenous G genotype isolated from a case of AFP, is a pathogenic strain with a tendency to cause inflammation.

Monocytes, dendritic cells, NK cells, and other cells when stimulated by IFN-γ secrete a chemokine IP-10, belonging to CXC family, mediating Th1 inflammatory response^30^. IP-10 levels were significantly increased in EV-A71-infected HFMD patients when compared with the healthy individuals^31^. IP-10 expression induced by EV-A71 infection increases IFN-γ and MIG levels, CD8 T cells infiltration, clearance of viruses in tissues leading to survival of mice^32^. Both circulating IFN-*γ*^21^ and IP-10 levels were increased in patients with EV-A71 PE (Pulmonary Oedema). Similarly in our study significantly elevated IP-10 release has been observed in neuronal cells infected with G and C genotypes but not in genotype D infected cells up to 24 hpi. However D genotype virus could induce IP10 secretion at 48 hpi. Further, the expression of IP10 gene induced by all three genotypes could be confirmed by real-time PCR. Although the secretion of IP10 and IFN-γ are inter-related, very low levels of IFN-γ could be induced by C genotype at 24 hpi and by the G genotype at 48 hpi. Previous studies suggested that IFN-γ may be involved in the progress of EV-A71caused severe complications^21^ and also showed that the changes of the serum levels of IFN-γ in severe patients were different, in which the levels of IFN-γ were very low in the first day and then increased for the first three days and then decreased^33^.

In EV71 infection, severely infected patients (including brainstem encephalitis, neurogenic pulmonary oedema, and sepsis) showed significantly higher TNF-α than those with mild infection indicating involvement of TNF-α in disease severity^33–35^. In our study significantly elevated TNF-α levels could be estimated from the G and C (Enc) infected cells corroborating the published reports but not by the C (HFMD) and D genotype infected neuronal cells at 24 hpi, confirming the pathogenicity of the G and C (Enc) strains. However all the four viruses induced TNF-α at 48 hpi.

The study was conducted in vitro with human cell lines due to the unavailability of clinical samples of HFMD cases since no recognizable-size outbreaks of EV-A71 HFMD, AFP or encephalitis have been reported in India, although the virus is endemic. The purpose of the study was to explore the tendency of the indigenous strains to infect human cells, therefore we have restricted the cytokine studies to certain cytokine/chemokines which are widely reported for EV-A71 infection, not the whole range of pro-inflammatory cytokines. We could not detect IFN-γ and TNF-α release at the early time point (12 hpi) in EV71 infected neuronal cells.

In summary, we found that indigenous EV-A71 genotype G, isolated from the stool of an AFP patient could induce highest expression of cytokine/chemokine genes and secretion of majority of the inflammatory cytokines tested. Most importantly the D genotype which has been isolated from both healthy and AFP cases in India (Saxena et al., unpublished) is not capable of inducing inflammatory cytokines up to 24 hpi. Although the C genotype viruses (isolated from CSF/vesicular fluid) and the indigenous genotype G virus (isolated from AFP case) showed significantly elevated pro-inflammatory cytokine/chemokine secretion in human neuronal cells confirming their pathogenicity, so far no EV-A71 caused outbreak or epidemic of HFMD have been reported in India. The D genotype has been isolated from different states of India in number of studies by our institute such as Gorakhpur (UP), Tripura, Haryana, Mumbai etc. and therefore reported to be most widely circulated. In addition, a sero-prevalence study conducted by us has shown higher antibody titres against EV71-D confirming the wide circulation of this genotype (manuscript communicated). Therefore we presume that Indian population might have been exposed to EV-A71 genotype D, which is acting as an attenuated virus to develop population immunity to resist pathogenic EV-A71 (genotype C and G) infection. Besides this the host genetic factors such as HLA types and proteins such as IRES, ITAFs could be the reasons for protection towards EV71 infection in Indian population. Our study warrants further research and more systematic analysis to delineate the interaction between EV-A71 indigenous genotypes and host responses leading to protective immunity.

## Supplementary Information

Supplementary Figure S1.
