# Supplementary material for: Susceptibility and cytokine responses of human neuronal cells to multiple circulating EV-A71 genotypes in India
Source: Sci Rep. 2021 Sep 7;11:17751. doi: 10.1038/s41598-021-97166-x (PMC8423732; doi:10.1038/s41598-021-97166-x)
Supplement: Supplementary file 1 — Supplementary Figure S1. [file 41598_2021_97166_MOESM1_ESM.docx]

**Figure legend for Supplementary Figure S1:**

Virus binding assay by Flow cytometry: SK-N-SH cells were mock-infected or infected with EV-A71genotypes at an MOI of 10 for 4 hrs. Cells were probed with anti-EV71 monoclonal antibody and anti-mouse FITC and analysed by BD-FACS-Canto II flow cytometer. Three independent experiments were performed, representative bar diagram of one experiment shown.

**Supplementary Figure S1**
